# O010: A novel immediate pre-operative decolonization strategy reduces surgical site infections

**DOI:** 10.1186/2047-2994-2-S1-O10

**Published:** 2013-06-20

**Authors:** E Bryce, T Wong, D Roscoe, L Forrester, B Masri

**Affiliations:** 1 Medical Microbiology and Infection Prevention and Control, Vancouver Coastal Health, Vancouver, Canada; 2 Quality and Patient Safety, Vancouver Coastal Health, Vancouver, Canada; 3 Department of Surgery, Vancouver Coastal Health, Vancouver, Canada

## Introduction

Pre-operative decolonization therapy (DcTx) using chlorhexidine (CHG) body washes and/or intranasal mupirocin can reduce surgical site infections (SSI) but compliance is often suboptimal. The effectiveness of a novel approach to immediate pre-operative decolonization therapy using intranasal antimicrobial photodisinfection therapy (PDT) and CHG body wipes in reducing SSIs was assessed.

## Objectives

To determine if immediate pre-operative decolonization using PDT and CHG body wipes reduced SSI rates.

## Methods

Between Sept 1, 2011 and Aug 31st 2012, 3068 elective cardiac, orthopedic, spine, vascular, thoracic and neurosurgery patients were treated with CHG wipes the night prior and day of surgery and received intranasal PDT in the preoperative waiting area. Weekend cases, procedures performed after the pre-operative dayshift, and emergency cases going directly to the operating room were not eligible. SSI surveillance remained unchanged from previous years and patients were followed for a minimum of three months. Results were compared to a historical control group consisting of 12,387 patients over four years and to a concurrent control group of 196 untreated patients.

## Results

A significant reduction in the SSI rate was observed after the intervention [historical-control group 2.7% and treatment group 1.6% (p <0.0001 RR 1.0114)]. The risk of having a *Staphylococcus aureus* infection was higher in the concurrent untreated (61%= 11/18 infections) compared to the treated group (32%=16/50 infections). The reduction in SSIs compared to the historical rates resulted in a cost avoidance of approximately $1.2 Million (Can) and would have permitted approximately 140 additional surgeries to be performed.

## Conclusion

The combination of PDT and CHG wipes immediately pre-operatively reduces SSIs and is cost-effective.

## Disclosure of interest

E. Bryce Grant/Research support from The Vancouver Hospital Foundation funded this project. Ondine Biomedical discounted the cost of PDT supplies and provided technical advice but had no role in data collection, analysis or interpretation of results, T. Wong Grant/Research support from The Vancouver Hospital Foundation funded this project. Ondine Biomedical discounted the cost of PDT supplies and provided technical advice but had no role in data collection, analysis or interpretation of results, D. Roscoe Grant/Research support from The Vancouver Hospital Foundation funded this project. Ondine Biomedical discounted the cost of PDT supplies and provided technical advice but had no role in data collection, analysis or interpretation of results, L. Forrester Grant/Research support from The Vancouver Hospital Foundation funded this project. Ondine Biomedical discounted the cost of PDT supplies and provided technical advice but had no role in data collection, analysis or interpretation of results, B. Masri Grant/Research support from The Vancouver Hospital Foundation funded this project. Ondine Biomedical discounted the cost of PDT supplies and provided technical advice but had no role in data collection, analysis or interpretation of results.

